# Tissue-specific atlas of trans-models for gene regulation elucidates complex regulation patterns

**DOI:** 10.1186/s12864-024-10317-y

**Published:** 2024-04-17

**Authors:** Robert Dagostino, Assaf Gottlieb

**Affiliations:** McWilliams School of Biomedical Informatics, University of Texas Health Science Center at Houston, Houston, TX USA

**Keywords:** Transcriptional regulation, Transcriptome imputation, Transcription factor polymorphism

## Abstract

**Background:**

Deciphering gene regulation is essential for understanding the underlying mechanisms of healthy and disease states. While the regulatory networks formed by transcription factors (TFs) and their target genes has been mostly studied with relation to *cis* effects such as in TF binding sites, we focused on *trans* effects of TFs on the expression of their transcribed genes and their potential mechanisms.

**Results:**

We provide a comprehensive tissue-specific atlas, spanning 49 tissues of TF variations affecting gene expression through computational models considering two potential mechanisms, including combinatorial regulation by the expression of the TFs, and by genetic variants within the TF.

We demonstrate that similarity between tissues based on our discovered genes corresponds to other types of tissue similarity. The genes affected by complex TF regulation, and their modelled TFs, were highly enriched for pharmacogenomic functions, while the TFs themselves were also enriched in several cancer and metabolic pathways. Additionally, genes that appear in multiple clusters are enriched for regulation of immune system while tissue clusters include cluster-specific genes that are enriched for biological functions and diseases previously associated with the tissues forming the cluster. Finally, our atlas exposes multilevel regulation across multiple tissues, where TFs regulate other TFs through the two tested mechanisms.

**Conclusions:**

Our tissue-specific atlas provides hierarchical tissue-specific *trans* genetic regulations that can be further studied for association with human phenotypes.

**Supplementary Information:**

The online version contains supplementary material available at 10.1186/s12864-024-10317-y.

## Background

Transcription regulation plays a key role in immune response [1] and in a broad range of diseases [2]. Understanding the gene regulation plan has the potential to help in identifying disease etiology and designing therapeutics. Importantly, previous studies suggest that gene regulation is tissue specific, driven by context-dependent regulatory paths, providing transcriptional control of tissue-specific processes [3, 4].

One of the methods to unravel the connection between genotype and transcription levels is through transcriptome imputation techniques, such as PrediXcan or TWAS [5, 6]. These methods model the genetic component of observed gene expression using combinations of genetic locations in *cis* with the gene they impute. The use of transcriptome imputation techniques have proven valuable in several scenarios, including complex human disease research and identification of trans-acting components [7–12], but these *cis* models still explain only a small portion of the variation in gene expression. Indeed, one study estimated that most heritability is driven by weak trans-eQTL single-nucleotide polymorphism (SNPs), supporting an approach to identify such sources of expression variability [13]. Two data-driven studies to identify *trans* effects were based on the *cis*-transcriptome imputation technique of PrediXcan. The first one learned a model for every pair of source and target genes, but it suffered from low power due to the large hypothesis space, which resulted in low number of discovered relationships [12]. They also tested their approach on either a single tissue (whole blood), or included all the tissues into one model, thus missing tissue specific information. The second approach looked for correlation between proteomics and the imputed gene expression, leading to both *cis* and *trans* correlations [14]. While this approach tested all 49 tissues, it was against proteomics from a single tissue. Additionally, both approaches suffered from two additional limitations: 1) it only tested one *trans* gene at a time, potentially missing combinations of *trans* effects; and 2) the data-driven approach leaves the potential mechanisms of these discovered *trans* effects determined.

To address the missing explained variance in *cis* transcription imputation methods and to overcome the low power and limitations of the aforementioned studies, we noted that both approaches found that *trans*-acting genes were enriched in transcription binding pathways and target genes were enriched in known transcription factor binding sites [12, 14]. Correspondingly, we previously introduced a new hypothesis-driven approach to generate trans-association models. Instead of looking at the entire genome, we focused on associations between variations in transcription factors (TFs) and the transcription levels of their transcribed genes [15]. We developed computational models accounting for two potential mechanisms whereby the combined variability of TFs can affect the expression of a transcribed gene. One mechanism considers variations in the expression of TFs affecting the expression their transcribed genes. Another mechanism suggest that deleterious single nucleotide polymorphisms (SNPs) within TFs may affect binding affinity, which leads to altered transcription levels of the transcribed genes.

The previous publication focused on methodology and was demonstrated only on two tissues [15]. The purpose of this study is therefore to provide an atlas of TF expression models for 49 different tissues and characterize their traits. We demonstrate through similarity between tissues that our discovered genes corresponds to other genomic types of tissue similarity. We further elucidate enrichment of modeled genes with pharmacogenomic phenotypes and with cancer and metabolic pathways. Clustering the tissues based on shared genes, we demonstrate that common genes are enriched with immune system regulation while cluster-specific genes are associated with phenotypes associated with the same tissues in which they were discovered. To allow for exploration of the regulation networks formed by our models, we provide a website (https://tstr.uth.edu).

## Results

### Distribution of genes modeled by tissue-specific TF models

**Fig. 1:**
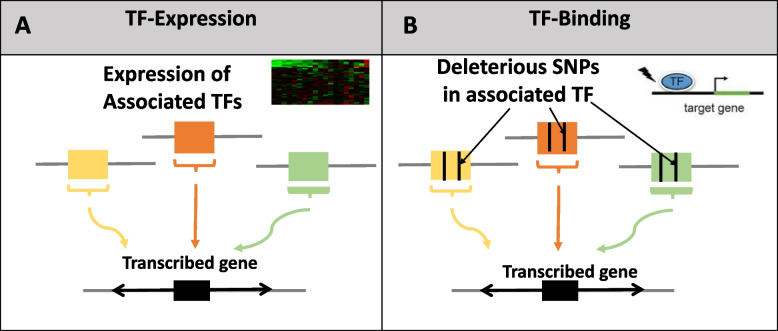
An illustration of the two tested mechanisms regarding the assocation between variabiliy in TFs and expression of their transcribed genes. TF-expression model includes associations of TF expression with estimated *trans* gene expression (**A**); and TF-binding model includes association of deleterious SNPs within the associated TF with estimated *trans* gene expression (**B**)

**Fig. 2:**
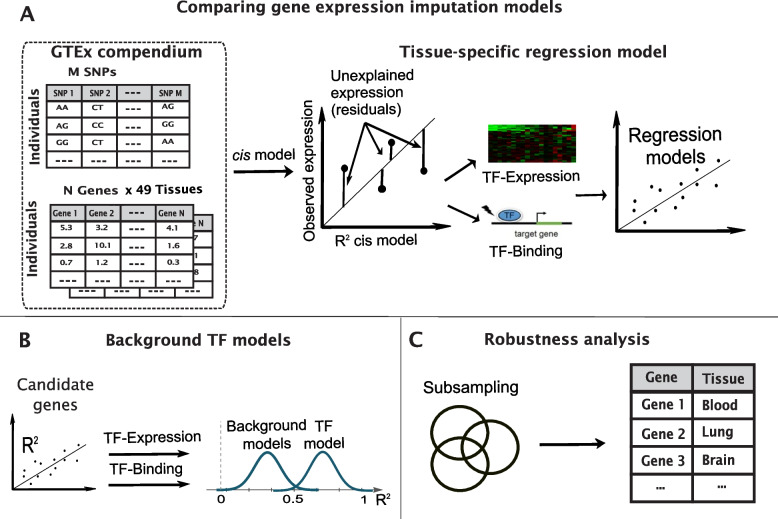
An illustration of the pipeline to identify hit genes. We compute the estimated *trans* GReX on the residulas after removing *cis* genetic effects and other effects on gene expresssion (**A**), test their significance relative to background models (**B**), and conduct a robustness test to validate the results (**C**)

We considered two potential mechanisms by which variability in TFs is associated with variability in expression of their transcribed genes: (1) the combined expression levels of TFs is associated with expression of their transcribed gene (Fig. 1A); or (2) combination of deleterious SNPs within the TFs is associated with expression of their transcribed gene (Fig. 1B). We refer to the computational models capturing these potential mechanisms as TF-Expression (mechanism number 1) and TF-Binding (mechanism number 2), respectively (Methods, Fig. 2).

Applying our models to 49 tissues and cells from GTEx, we extracted “hit genes” for which the TF-Expression and/or TF-Binding models passed the significance tests (explain more variance than a random model) and robustness test (consistently discovered with subsets of the samples) (Methods). We discovered a total of 6,147 hit genes using the TF-expression model (average 295 ± 93 hit genes per tissue) and 6,265 hit genes using the TF-binding model (182 ± 60 hit genes per tissue) (Table S1, Figure S1). The majority of the discovered genes were tissue-specific, with each gene discovered in 2.4 ± 3.5 tissues on average for the TF-Expression model and in 1.4 ± 0.75 tissues for the TF-Binding model (Figure S2).

We measured the correlation between the R^2^ of explained expression variance between *cis* (PrediXcan) models and our models across tissues. The TF-Expression model showed moderate negative (ρ = -0.22, *p* < 6e^−165^), While the TF-Binding model displayed negligible correlation (ρ = -0.03, *p* < 0.003, Figures S3-S4).

### Model-based tissue similarity is comparable to other tissue similarity metrics

To augment our previous validation of the methodology in two tissues [15], we validated the models across the entire 49 tissues and cells. We compared tissue similarity based on the modelled genes to tissue similarity computed based on expression and tissue-shared genetic regulation based on gene-level profiles of DNase I hypersensitive sites (DHS) of Zhou et al. [16] (Methods).

When compared to Zhou et al. tissue similarity that is based on gene expression profiles, we found a significant correlation to our TF-Expression shared genes similarity (Spearman ρ = 0.43, *p* < 2e^−54^, Figure S5). Since the comparison is to an expression-based tissue similarity, the comparison to the TF-Binding model was lower but still statistically significant (Spearman ρ = 0.17, *p* < e^−8^, Figure S6).

When compared to tissue similarity based on DHS [16], we found good correspondence between the DHS similarity and the similarity computed on the genes from both the TF-Expression model (Spearman ρ = 0.47, *p* < 4e^−65^, Figure S7) and the TF-Binding model (ρ = 0.25, *p* < 7e^−18^, Figure S8).

### Characterization of hit genes

We first characterized the hit genes discovered through our TF modes by testing the hit genes for conservation using the LIST [17] conservation scores (Methods). We found that hit genes were significantly more conserved relative to genes that did not pass the model filtering in both the TF-Expression (Wilcoxon ranked sum test, *p* < 2e^−15^) and in the TF-Binding models (*p* < e^−69^).

Inspecting individual tissues, all individual tissues in TF-Binding had higher conservation scores than the background. For the TF-Expression model, forty tissues (out of 49) obtained higher conservation scores than the background. The nine tissues that obtained lower conservation scores than the background score include brain tissues, colon and esophagus.

Another characteristics is that both the TFs participating in the models and the modeled hit genes in both TF-Expression and TF-Binding models were enriched for pharmacogenes – genes with variants associated with pharmacogenomic traits from PharmGKB [18] (*p* < 3e^−9^ and *p* < 9e^−9^ for TFs in TF-Expression and TF-Binding models, respectively and *p* < 6e^−21^ and *p* < 3e^−9^ for hit genes in TF-Expression and TF-Binding models, respectively).

### Hit genes common to multiple tissues are enriched with immune response

We next looked for identifying traits for hit genes discovered across at least half of the tissues (Methods). We identified 56 common hit genes in the TF-Expression model and four common hit genes (ANKRD65, POLR1A, VPS28, NDUFV1) in the TF-binding model (Table S1 display the number of tissues per hit gene).

The 56 common genes in the TF-Expression models were enriched with multiple Gene Ontology (GO) biological process terms, including general immune systems terms such as “innate immune response”, “regulation of immune system process” and “defense response to other organisms” (B&H FDR adjusted *p* < 4e^−10^), and more specific immune response-related GO terms include activation of myeloid leukocytes, neutrophils and granulocytes (*p* < 5e^−6^). Additional enrichments are for Reactome pathways related to the immune system, including “cyclic GMP-AMP synthase (cGAS)-stimulator of interferon genes (STING) mediated induction of host immune responses” (B&H FDR adjusted *p* < 0.005) and Reactome’s “diseases of immune system” (*p* < 0.02, Table S2). We highlight one gene of the 56 common genes, CD33, a microglial inhibitory Siglec [19]. In our set, CD33 was discovered in several brain tissues, including brain cerebellum, frontal cortex, hippocampus, putamen and caudate basal ganglia. In these tissues, the TF with the highest weight in the model is the gene SPI1 (PU.1). Interestingly, higher CD33 expression in the parietal lobe is associated with advanced cognitive decline or Alzheimer’s disease status [19–21]. Furthermore, In support of this finding, the TF SPI1 is also reported to regulate disease-associated genes in primary human microglia [22] and expression levels regulate microglial inflammatory response [23].

Out of the four common hit genes in the TF-Binding model, we highlight two, RNA Polymerase I Subunit A (POLR1A) and Mitochondrial complex I deficiency (NDUFV1). POLR1A was modeled in ten tissues, including multiple tissues from the gastrointestinal tract (sigmoid and transverse colon, esophagus mucosa, small intestine terminal ileum, stomach and minor salivary gland). Inhibition of POLR1A was found to regulate the signaling pathways and cell functions in colorectal cancer [24, 25], so its trans-regulating TFs may also be involved. There are several TFs in their models across these digestive system tissues, but the most highly weighted TFs are RNA Polymerase I Subunit A (POLR1E) and genes from the Signal Transducer And Activator Of Transcription family STAT2, STAT3 and STAT4. Indeed, POLR1E is part of enriched signaling pathways in colorectal cancer [26, 27] while these member of the STAT family are contributing to promotion of colorectal tumorigenesis, silencing them inhibits cell proliferation and invasion of colorectal cancer cells, and considered as targets for therapy [27–34].

NDUFV1 was primarily discovered in 11 tissues, seven of which are brain tissues (amygdala, caudate basal ganglia, cerebellum, cortex, hypothalamus, putamen basal ganglia and substantia nigra). Genetic variations and differential expression of NDUFV1 are associated with several human neurological disorders, including Mitochondrial Complex I Deficiency, Parkinson disease (PD), Alzheimer’s Disease (AD), myoclonic epilepsy, schizophrenia, Leigh syndrome and leukoencephalopathy [35–45]. The brain tissue models are prevalent with three TFs, ETS Proto-Oncogene 1, Transcription Factor (ETS1), Forkhead box protein O1 (FOXO1) and PR/SET Domain 14 (PRDM14), each appearing in almost half of the brain tissues. These TFs are also associated with some of the same neurological conditions associated with regulation of their modeled hit gene, NDUFV1: ETS1 is associated with complex I deficiency [42, 46], expression levels of FOXO1 are decreased in acute schizophrenia [47] and increased in AD [48]. Finally, PRDM14 has key role in modulating specific regulatory functions in schizophrenia [49], suggesting a possible mechanism for these three TFs to affect these conditions through NDUFV1.

### Tissue cluster-specific gene modules

**Fig. 3:**
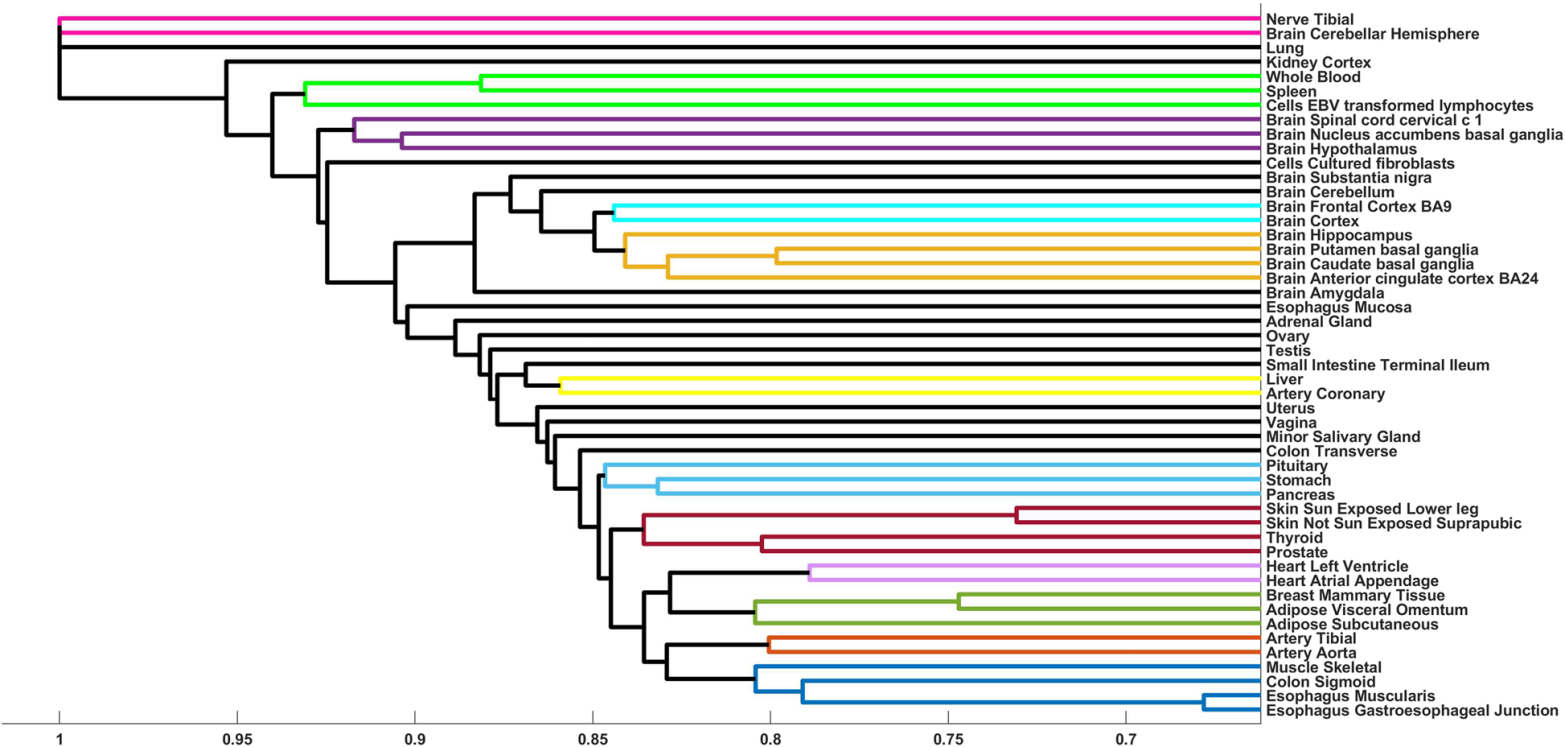
Dendrogram of the clustered tissues based on hit genes discovered in the TF-Expression model. Each cluster is in separate color, with black lines denoting signleton clusters

**Fig. 4:**
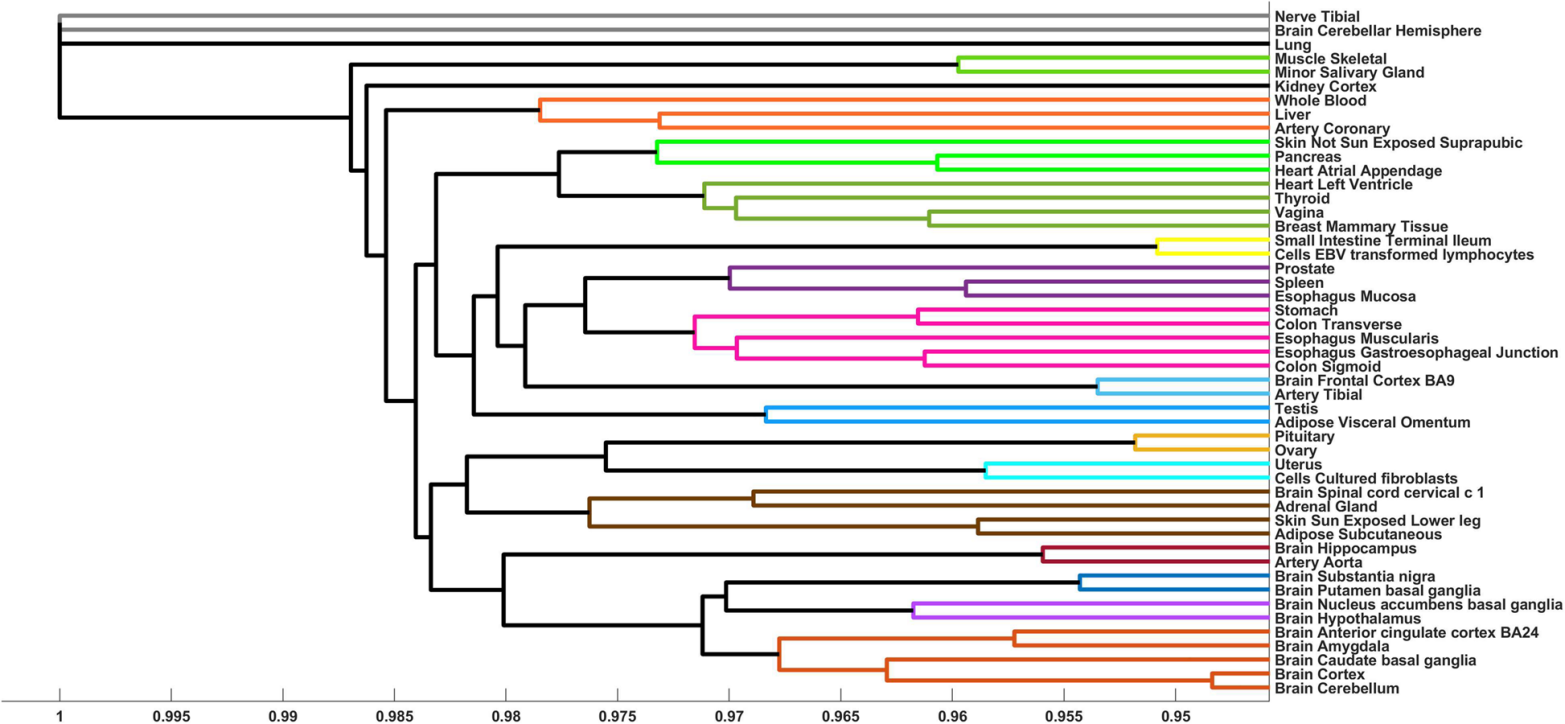
Dendrogram of the clustered tissues based on hit genes discovered in the TF-Binding model. Each cluster is in separate color, with black lines denoting signleton clusters

We were interested in tissue-specific hit genes but in order to account for similar tissues, we first clustered the tissues based on the hit genes, choosing hierarchical clustering to reflect the tiered nature of tissues, such as organs and organ systems (Methods). We identified 12 clusters from the TF-Expression model and 17 clusters from the TF-Binding model, not including tissues that form singleton clusters. Several of these clusters grouped tissues from the same organ or organ system, such as brain, gastrointestinal, adipose, skin, or heart tissue clusters (Figs. 3 and 4).

The majority of the clusters do not overlap between the TF-Expression and TF-Binding models. Notable examples of clusters that are shared between these models include tissues belonging to the digestive system, including sigmoid colon, esophagus gastroesophageal junction and esophagus muscularis, brain tissues (anterior cingulate cortex ba24 and brain caudate basal ganglia), and a cluster grouping liver and coronary artery (Figs. 3 and 4).

We identified 199 cluster-specific hit genes across all clusters in the TF-Expression and 263 cluster-specific genes in the TF-Binding model (Methods). We highlight here two such examples. The first example involves a cluster of digestive system tissues in the TF-Expression model. This cluster has three cluster-specific genes, GOLT1A, RAB27B and SLC28A3. GOLT1A has been reported to be differentially expressed between high and low risk groups for esophageal cancer [50] and RAB27B is a significant prognostic marker for metastasis and poor prognosis in colorectal cancer [51–53]. The TF that appears in the models of all three hit genes is GATA-binding factor 2 (GATA2). GATA2 is highly expressed in colorectal cancer cells and serves a prognostic factor [54–56].

The second example is a cluster from a TF-Binding model, including brain hypothalamus and nucleus accumbens basal ganglia tissues. There are six cluster-specific genes (PRKCA, GPNMB, ADI1, KCNMB4, CCZ1B, and SLC13A3) and these genes are enriched for GO annotation of regulation of neurotransmitter secretion (*p* < 0.05), consistent with the brain tissues where they were modeled [57, 58]. The two prominent TFs in their models are TFAP2A and MXI1, where noradrenergic neurons require TFAP2A for expression of a neurotransmitter phenotype and promote specification and maturation of neurons, while MXI1 is essential for neurogenesis and acts by bridging the pan-neural and proneural genes [59–63].

### Co-regulation of hit genes

We tested for co-regulation of genes by identifying pairs of hit genes that co-discovered across several tissues (Methods). We identified 1,157 co-regulated hit genes pairs in the TF-Expression model, and only 23 gene pairs from the TF-Binding models, corresponding to a larger portion of tissue-specific hit genes modeled by the TF-Binding model. The majority of the co-regulated gene pairs in the TF-Expression are in one connected module (77% of the pairwise connections). We provide visualization of these co-regulation networks on our dedicated website (https://tstr.uth.edu).

**Fig. 5:**
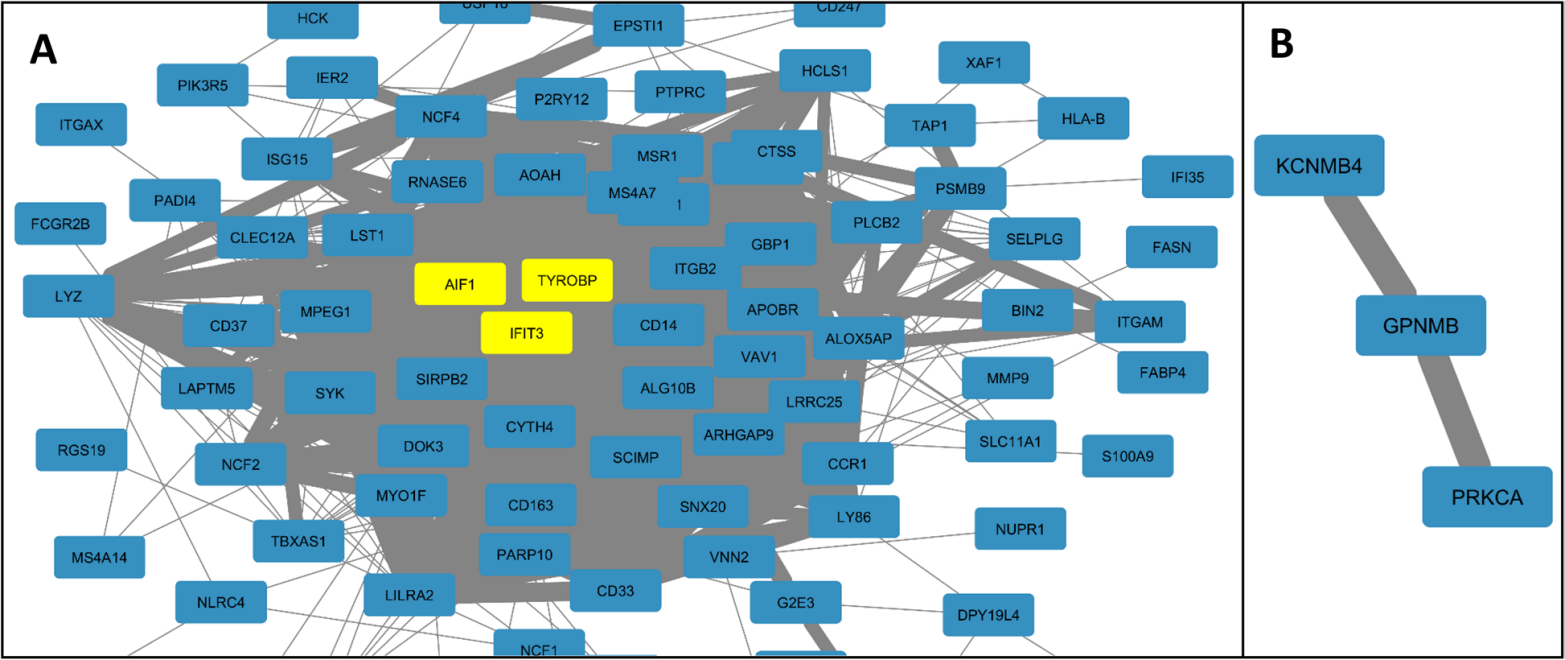
Discussed examples of co-regulation from TF-Expression model (**A**), where the discussed examples are in yellow and their direct neighbors in blue; and TF-Binding model (**B**)

We highlight two examples of prevalent co-regulation (Fig. 5). The first example from the TF-Expression involves three genes, TYROBP (DAP12), IFIT3, and AIF1 (IBA1), which were discovered in the TF-Expression model across more than 37 shared tissues (Jaccard score > 0.8, *p* < 0.01). Interestingly, all three genes have increased expression in microglia [64, 65] and are part of a gene signature in a mouse model of amyotrophic lateral sclerosis, where expression of IFIT3 and TYROBP increased during disease progression in the mice microglia, macrophages, neutrophils, and monocytes [66].

The second highlighted example is from the TF-Binding model, involving the genes PRKCA, GPNMB and KCNMB4. These genes were discovered across various brain tissues (cortex, hippocampus, hypothalamus and nucleus accumbens basal ganglia). Microglia express GPNMB in the brains of Alzheimer's disease and Nasu-Hakola disease [67], PRKCA is associated with neural basis of episodic remembering in healthy individuals [68] and KCNMB4 is downregulated in hippocampal granule neurons following seizure activity [69]. The three TFs that have the highest cumulative weight in the models of these genes are SREBF1, SREBF2 and ZEB1, themselves associated with neuronal differentiation and neurodegenerative diseases like Alzheimer’s Disease or Huntington’s Disease [70–72].

### Regulation cascades of hit genes and TFs

We identified regulation cascades, where a gene’s expression is modeled by the TFs in the model, and in turn, some of these TFs are also modeled by other set of TFs, forming regulation cascades in specific tissues. In order to enable exploration of these regulation cascades in each tissue, we’ve provided a web server (https://tstr.uth.edu/), showing regulation cascades that reach up to four levels of *trans*-regulation (in the small intestine and in the aortic artery tissues in the TF-Expression model).

**Fig. 6:**
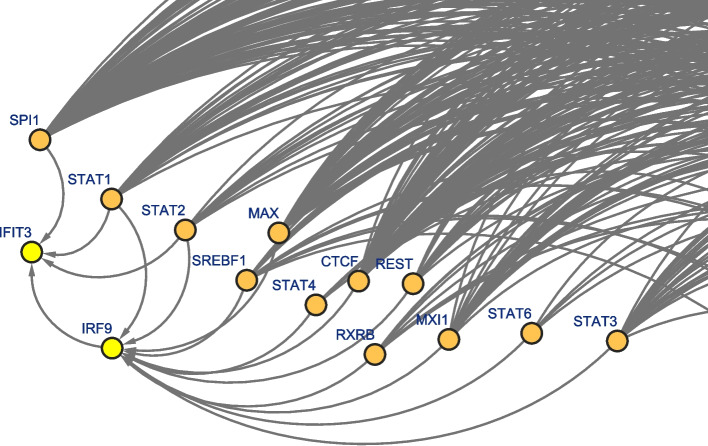
Example of regulation cascade discovered in the TF-Expression model in Mammary breast tissue between IFIT3 (hit gene, in yellow), IRF9 (both TF and hit gene, yellow). Other TFs, such as STAT1 and STAT2, are included in the models for IFIT3, IRF9 or both (orange)

We highlight one such regulation cascade: Interferon-induced protein with tetratricopeptide repeats 3 (IFIT3) is a hit gene that is modeled in 38 tissues by the TF-Expression model, each involving different combinations of TFs affecting IFIT3. Four TFs, Signal Transducer And Activator Of Transcription 1 and 2 (STAT1 and STAT2), and interferon regulatory factor 1 and 9 (IRF1 and IRF9) are not only prevalent across these models, but they are also modeled themselves by the TF-Expression model across nine different tissues where IFIT3 is also modeled. Furthermore, in four tissues such as breast and esophagus mucosa, both IFIT3 and IRF9 are modeled by STAT1 and STAT2, suggesting a high level of regulation (Table 1, Figs. 6, S9-S11). This regulation pattern is supported by previous publications, demonstrating that STAT1–STAT2–IRF9 form complexes, known as IFN-stimulated gene (ISG) factor 3 complexes [73, 74]. Furthermore, high expression of STAT1, STAT2, and IRF9 in breast cells significantly increase the expression of IFIT3 after IFNβ treatment [75], are all highly expressed in cells such as esophageal squamous cell carcinoma [76, 77] and that STAT2 could form a complex with IRF9 and bind to the IFN-stimulated gene regulatory element (ISRE) sequence on the IFIT3 promoter to promote IFIT3 transcription [78]. In another publication regarding patients with Chronic Hepatitis B Virus, STAT2 was essential for the production of IFIT3 but not STAT1 [79].

**Table 1:** Regulation cascade of TFs modeling IFIT3 and also modeled themselves

Tissue	IRF1	IRF9	STAT1	STAT2
Adipose subcutaneous			X	
Adipose visceral omentum			X	
Brain amygdala		X		
Brain spinal cord cervical c-1			X	
Breast mammary tissue		X		
Esophagus mucosa		X		
Heart atrial appendage				X
Skin sun exposed lower leg		X		
Whole blood	X

We highlight the possible co-regulation of IFIT and its TF IRF9. IRF9 has the highest weight in the models of IFIT3 and is modeled in four tissues where IFIT3 is also modeled (Table 1). One of these tissues is breast. Recent studies have implicated IFIT proteins as prognostic markers to determine the clinical outcome of breast cancer [80]. IRF9 is not only associated with the development of resistance to antimicrotubule agents in breast tumor cells, but is also reported as potential link to downstream mediators of IFN signaling to drug resistance in human cancers [81]. Another tissue where the signaling cascade of interferon is involved is sun exposed skin (Table 1). Indeed, there is prognosis and biological significance for IFIT family in skin cutaneous melanoma and is a novel regulatory factor in psoriasis [82, 83]. Additionally, healthy primary keratinocytes increase interferon response genes, including IRF9 [84]. Although STAT1 and STAT2 are not hit genes by themselves, TFs from the STAT family have established connections to breast cancer and skin [29, 84–87]. It is interesting to note that in breast and amygdala tissues, both STAT1 and STAT2 participate in the TF-Expression model for IRF9 (Figs. 6, S9), but in the esophagus only STAT2 is included in IRF9 model (Figure S10) and for skin only STAT1 is included, which could be related to the accumulating evidence revealing that distinct facets of STAT2 and IRF9 activity mediated by the segregation in alternative STAT1-independent complexes are thought to trigger different transcriptional programs [88].

## Discussion

Transcriptome imputation methods focus on *cis* areas of the genes and suffer from missing explained variance in gene expression. We introduced models for explaining the missing variance by considering *trans* effects of variability in TFs on gene expression in the form of transcriptional variation (TF-Expression) or genetic variation (TF-Binding) in TFs. Here, we applied our models to 49 tissues and cells, characterized the thousands of discovered hit genes affected by variation in TFs across the tissues (6,147 and 6,265 hit genes in each model), and generated an atlas of this type of regulation.

The majority of the discovered hit genes are specific to a single tissue, or few related tissues, which corresponds well to the observation of Lopes-Ramos et al. [89] that TF regulation is tissue specific. This is mostly apparent in the TF-Binding model, where no hit gene was common to more than five tissues, while some of the hit genes in the TF-Expression model were common to more than twenty tissues. We hypothesize that since TFs typically transcribe several genes, major changes in TF binding may result in significant effect to several genes and cellular processes, which could be detrimental to the individual. Such genetic variation may suffer from evolutionary pressure to attenuate its effect. In contrast, affecting the regulation through expression variation of the TF can be more fine-tuned. Furthermore, the overlap of genes between the two models, TF-expression and TF-binding, in each tissue do not exceed 2.5%, suggesting that these mechanisms are complementary in each tissue.

A small subset of the genes in the TF-Expression are common to multiple tissues (56 genes). These genes are enriched for multiple GO terms and diseases related to the immune response, suggesting that these immune functions are prevalent across multiple tissues and that these genes are regulated via expression variation of their corresponding TFs. This observation is in line with previous observation that genes involved in response regulation, such as TFs in our case, display a unique expression pattern across species and conditions, suggesting a fine-tuned regulation [90]. Additional support comes from the study of Wittich et al. [14] that observed that their target proteins of *trans*-acting genes were enriched for autoimmune diseases in the GWAS catalog. Given the enrichment of immune system hits in our TF-Expression models, we speculate that in the case of immune system, complex regulation plan that is not “hard-coded” into the genome but regulated by the transcription level of the TFs might provide more benefits. This type of immune-specific regulation complexity was previously discussed but quantification requires more research [91, 92].

We observed complex regulation cascades, where the TFs themselves were also regulated by other sets of TFs. These cascades were also tissue-specific. TF cascades were previously studied with regard to developmental gene networks [93], where these cascades were accurately timed, and are also prevalent in other model organisms [94, 95]. Further research into our hypothesized cascades is needed to determine if they occur in sequence in developed cells.

We list three limitations of our method. The first limitation is that it is hypothesis-driven and thus led us to focus on variability in TFs. This has allowed us to generate interpretable models and gain better understanding of TFs, which are components with well-established role in transcription regulation. However, we might have missed other *trans*-regulation components that can affect gene expression, and thus our models are likely to underestimate the *trans* effects on each gene.

A second limitation is specific to the TF-Binding models. We included only non-synonymous deleterious SNPs in these models with these two reasons in mind: 1) the biological interpretation of the models; and 2) the generalizability of the models. Specifically, the first reason concerns interpretability of our model, as variants predicted to be deleterious would be the initial suspects for affecting binding affinity while other variants, such as synonymous SNPs, would be harder to tie to the mechanism. The second reason is that incorporating a smaller and more focused set of variants helps address the high dimensional sparse space of variants, avoids overfitting of the models and enables reasonable computing time of these models. While dimensionality reduction techniques could be considered in subsequent works to incorporate non-deleterious variants into the models, these would aid computations time but may still reduce the interpretability and transferability of the models.

The final limitation is that our models identify only associations and not infer causation. Nevertheless, we anticipate that our findings will be useful in prioritizing genes and TFs for experimental setting that is designed to test their causality and relevance to specific conditions.

The deleterious variants included in the TF-Binding model could potentially affect both the binding domains and the structure of the TF, each with a potential to affect the binding affinity of the TF. Further research utilizing a large scale resource that differentiate between binding and structural domains within each TF could further determine the mechanistic interpretation of the model.

## Conclusions

Understanding how variations in TFs regulate gene expression can offer key insights into the transcriptional regulation plan. Correspondingly, we provide an atlas of computational models linking variations of TFs to gene expression levels of their transcribed genes across 49 tissues and make discovered co-regulation and regulation cascade networks available for examining through a dedicated website.

Our results can be utilized in two ways. First, our tissue-specific regulation models can be incorporated into genome wide association studies and improve phenotype prediction models. Second, our newly generated hypotheses for genetic regulation that can be further explored in the context of specific tissues through experiments.

## Materials and methods

### Data

Genotype and expression data from the Genotype-Tissue Expression Project (GTEx) version 8 [96] were retrieved from dbGaP for 49 tissues (Table S3). Transcription factors and their transcribed genes (204,999 unique gene-TF pairs) were assembled from three sources: Transcriptional Regulatory Relationships Unraveled by Sentence-based Text mining (TRRUST V2) [97], the Human Transcriptional Regulation Interaction Database (HTRIdb) [98] and the regulatory Network Repository of Transcription Factor and microRNA Mediated Gene Regulations (RegNetwork) [99]. Genomic positions of the TFs were computed based on the human genome assembly version 37 (GRCh37). Functional annotations for non-synonymous SNPs were retrieved from SnpEff v4.3 [100]. Evolutionary conservation scores were downloaded from LIST [17] and averaged across all the amino acids of each protein to obtain a protein conservation score. Pharmacogenomic clinical variants were downloaded from PharmGKB [18]. Pathways were retrieved from the Pathway Interaction Database [101] and The Reactome pathway knowledgebase [102].

### TF models for estimating trans associations with gene expression

To formulate our models, we start with the hypothesis, described in the PrediXcan method [6], that views the observed transcription levels of a gene, T_g_, as the combined effect of genetically regulated gene expression (GReX) and contribution of other factors, *ϵ,* assumed to be independent of the genetic component *cis* and *trans* effects of variants in the gene:

1 $${T}_{g}=GReX + \epsilon$$

We further split the estimate of *GReX*, $\widehat{GReX},$ to its combined *cis* and *trans* effects, ${GReX}_{cis}$, and ${GReX}_{trans}$. In this work we neglected interaction terms between *cis* and *trans* to simplify the models.

2 $$\widehat{GReX}={GReX}_{cis}+ {GReX}_{trans}$$

For estimating the *trans* effects, we model the residual genetic effect of ${\widehat{GReX}}_{trans}$ by subtracting the ${GReX}_{cis}$ component, predicted by PrediXcan method [6]*,* from a normalized expression value, that is adjusted for sex, the top 3 principal components (derived from genotype data) and the top 15 PEER factors [103], using the normalization procedures introduced in the PrediXcan paper:

3 $${\widehat{GReX}}_{trans}= {Tg}_{Norm}- {GReX}_{cis}$$

For modeling the genetic *trans* effects, ${\widehat{GReX}}_{trans}$, we used two hypothesized mechanisms:

- Model 1: A model assuming variability in the expression of the TFs affects variability in gene expression (TF-Expression). This model uses expression values of TFs as the independent variables:
  4 $${\widehat{GReX}}_{TF-expression}= {\sum }_{k}{\alpha }_{k}{T}_{k}+\epsilon$$

where *T*_*k*_ are the normalized TF expression level *and α*_*k*_ are the weights learned using the regularized LASSO regression [104] and *ϵ* is the contribution of other factors that determine the residual expression trait, assumed to be independent of the TF-expression component.

- Model 2. The second model assumes genetic variants in the TF gene affect the binding affinity to the transcription factor binding site (TF-Binding):
  5 $${\widehat{GReX}}_{TF-binding}= {\sum }_{k}{\beta }_{k}{V}_{k}+\epsilon$$

where β_k_ are the weights learned using the regularized LASSO regression and *V*_*k*_ are the dosages of SNPs within these TFs and *ϵ* is the contribution of other factors that determine the residual expression trait. For these models, we focused only on non-synonymous, deleterious SNPs, based on SIFT scores lower than 0.05 [105].

We tested each of these models independently so effects of each model does not mask another model.

### TF models construction

The constructed TF models follow a four-step pipeline that is also described in Lu et al. [15] (Fig. 2). We provide a brief description of these steps below:

- Step 1: Calculate residual variability unexplained by *cis*-models. We modeled the residuals between normalized observed genes expression and *cis*-imputed expression based on PrediXcan method [106]: The genetic component of the observed expression of each gene in each tissue was calculated using the normalization proposed in PrediXcan, accounting for gender, sequencing platform, the 3 top principal components of the genotype dosages, and the 15 probabilistic estimation of expression residuals (PEER) factors [106]. The result of the normalization was that the normalized gene expression closely resembled standard normal distribution (mean expression per gene 0.006 ± 0.97). The remaining residuals to model are obtained by subtracting the *cis*-imputed expression from the normalized expression.
- Step 2, build a model for each candidate gene: We model the residual genetic variability of the gene expression (dependent variable) using the regularized LASSO regression [104]. The dependent variables were either the normalized observed expression of the TFs, normalization of TF expression adjusting for the same variables as all the genes (TF-Expression model) or deleterious genetic variants within the TF gene (TF-Binding model).
- Step 3: Significance tests. In order to identify genes whose TF models are significantly different than random, we compared the computed R^2^ between the residual expression and the model prediction to R^2^ obtained from two sets of background models (each with a hundred runs) and retained only genes that passed both background models with significance level of FDR-adjusted p-value < 0.05 (Benjamini–Hochberg false discovery rate (B&H FDR) [107]). The first background model trained the models on shuffled residuals, while the second background model uses random selection of unassociated TFs. For the latter, we selected 100 random sets of TFs for each gene, each set with the same number of TFs as the true set of TFs associated with the gene.
- Step 4: Robustness test. We re-ran the entire pipeline (with background models) ten times, each on randomly selected 90% of the samples, retaining genes that were significant in more than 50% of the robustness tests, defined as “hit genes”.

### Comparison to similarity metrics

In Lu et al., [15] we validated hit genes in two tissue, skeletal muscle and whole blood, against eQTLs and a data-driven approach of Wheeler et al. [12]. Here, we validated our tissue-specific gene atlas by comparing a tissue similarity metric constructed from hit genes, to previously constructed tissue similarities based on other types of data. The comparison was done by calculating the Spearman correlation between our tissue similarity and the external tissue similarities across all tissues.

We computed our pairwise tissue-tissue similarity using Jaccard score between their shared genes in either the TF-Expression or TF-Binding models based on our discovered genes associated with variability in their TFs.

We compared our similarity metric to two types of tissue similarities introduced by Zhou et al*.* [16]: 1) an expression-based tissue similarity and 2) a tissue similarity capturing tissue-shared genetic regulation based on cell-specific gene-level profiles of DNase I hypersensitive sites (DHS). For the second metric, they mapped cells to tissues and provided normalized tissue similarity scores. The DHS-based tissue similarity is computed on the gene level (i.e., the similarity is provided between two tissues for each gene), so we calculated a tissue similarity by averaging across all the genes. Due to their method of standardization, the similarity provided by Zhou et al. is not symmetric, i.e., the average similarity between tissue A and tissue B (across all genes) is not the same as between tissue B and tissue A. We thus made it symmetric by averaging the similarity of each pair of tissue from both directions.

### Defining hit genes common to multiple tissues

We defined hit genes that are common across tissues by computing the maximum number of tissues that any hit gene appeared in (41 tissues in TF-Expression and 16 in TF-Binding), and selecting genes that appear in more than half of the number of these tissues (21 tissues and 8 tissues, respectively).

### Identifying tissue clusters

In order to gain insights into hit genes specific to a tissue group, we clustered the tissues using hierarchical clustering, capturing tissue hierarchy. We used average linkage that achieved highest cophenetic coefficient among linkage options (0.94 for the TF-Expression model and 0.68 for the TF-Binding model). We used median of the tree based on the inconsistency coefficient for each link of the hierarchical cluster tree [108] in order to decide on a cutoff to assign distinct clusters. We defined cluster-specific genes as hit genes that are predominant for a single cluster (FDR adjusted *p* < 0.05).

### Statistical analysis

Enrichments of pharmacogenes used hypergeometric test of hit genes relative to all the tested genes. Enrichments of hit genes within a cluster and hit genes within biological pathways were computed using hypergeometric test between genes in the cluster and genes in all clusters, and enrichment of hit genes with biological functions was computed using the ToppGene suit [109]. The co-regulation networks was computed across tissues. It includes genes that were modeled in at least three tissues and the tissue overlap between each gene pair has a Jaccard score of at least 0.5 and reached statistical significance (False discovery rate < 0.05). Statistical significance of these co-regulated genes was computed using hypergeometric test and all p-values were corrected using Benjamini–Hochberg false discovery rate [107].

### Supplementary Information

**Supplementary Material 1.**

## Data Availability

GTEx data is available from dbGaP (accession phs000424.v7.p2). Transcription factors and their transcribed genes are publicly available from Transcriptional Regulatory Relationships Unraveled by Sentence-based Text mining (TRRUST V2) [97], the Human Transcriptional Regulation Interaction Database (HTRI) [98] and the regulatory Network Repository of Transcription Factor and microRNA Mediated Gene Regulations (RegNetwork) [99]. The datasets generated and/or analyzed during the current study are available in: http://tstr.uth.edu. Code is freely available through GitHub: https://github.com/helu2008/transTFModel.
