# Supplementary material for: Tissue-specific atlas of trans-models for gene regulation elucidates complex regulation patterns
Source: BMC Genomics. 2024 Apr 17;25:377. doi: 10.1186/s12864-024-10317-y (PMC11022497; doi:10.1186/s12864-024-10317-y)
Supplement: Supplementary file 1 — Supplementary Material 1. [file 12864_2024_10317_MOESM1_ESM.zip › TF_map_supp_material.pdf]

Tissue-Specific Map of Trans-Models for Gene Regulation Elucidates Complex Regulation Patterns

Robert Dagostino [1] and Assaf Gottlieb [1,*]

[1] Center for precision health, McWilliams School of Biomedical informatics, University of Texas Health Science Center at Houston, Houston, Texas, United States of America

Figures

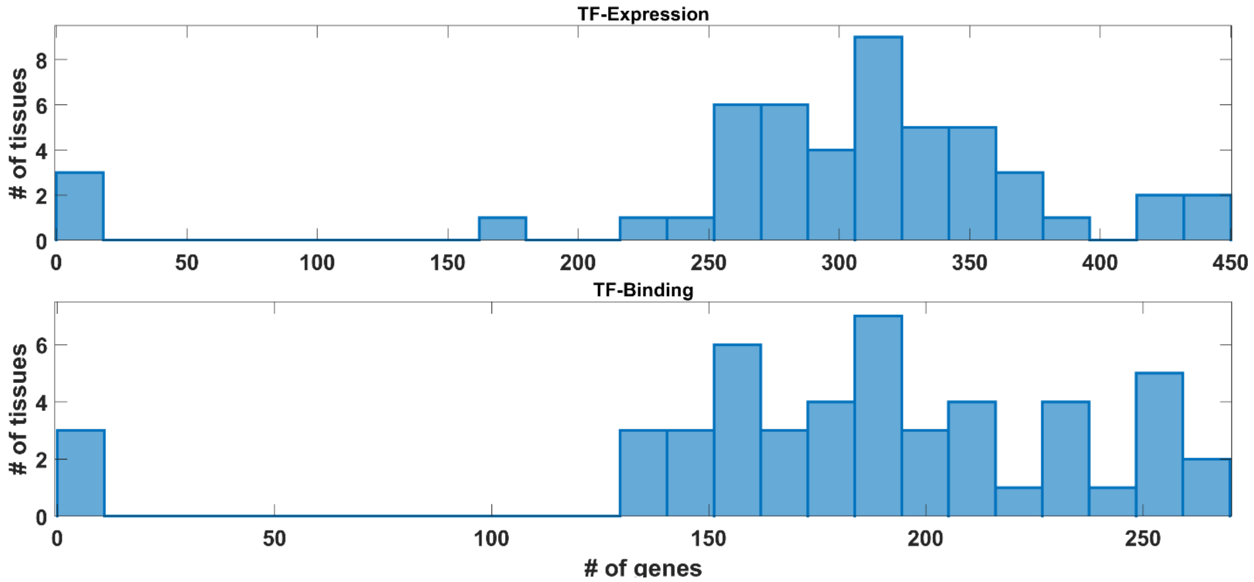

Figure S1. Histograms of the number of genes discovered in tissues for TF-Expression (Top) and TF-Binding (Bottom).

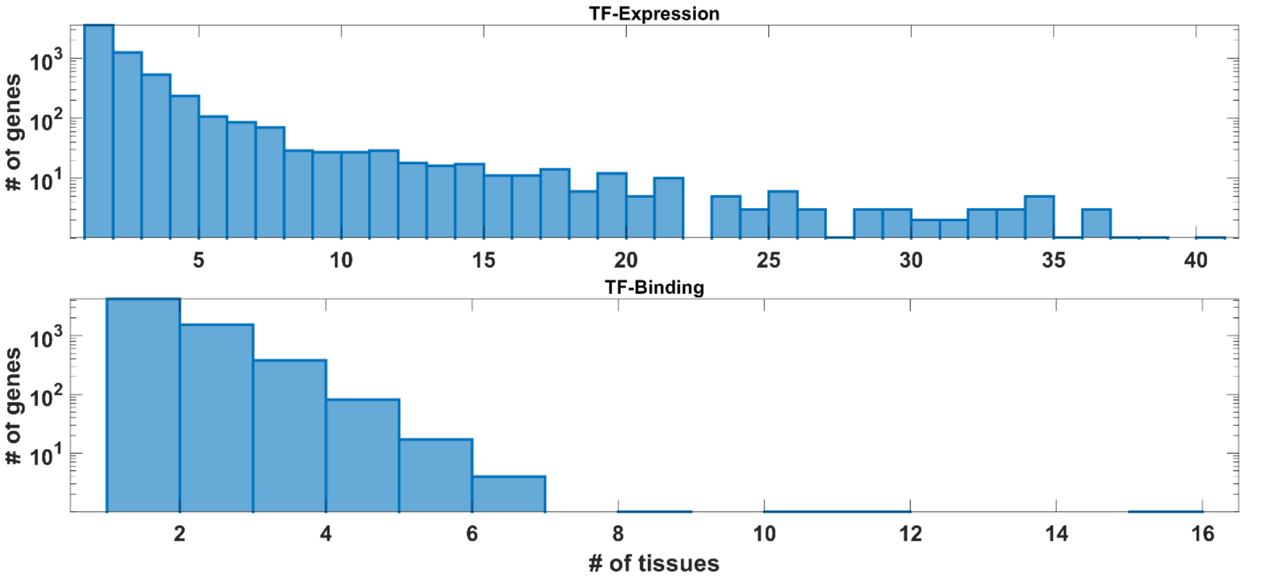

Figure S2. Histograms of the number of tissues per gene for TF-Expression (Top) and TF-Binding (Bottom). y-axis is log-scaled.

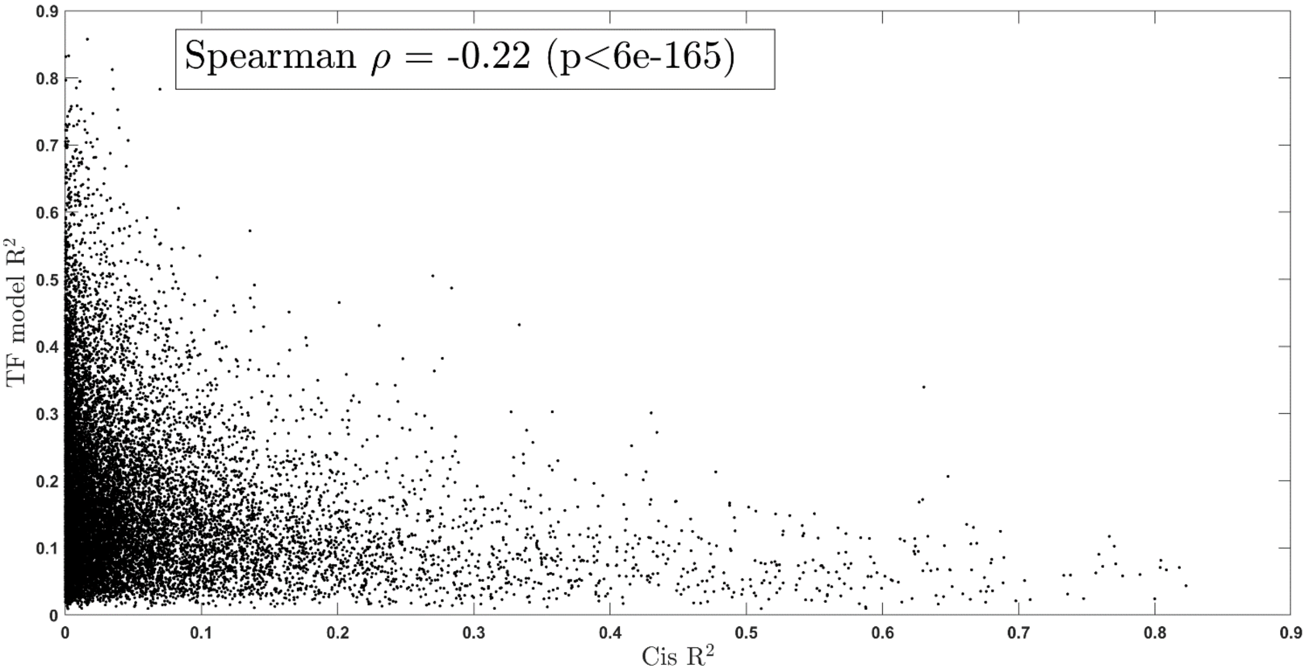

Figure S3. Scatter plot of the TF-Expression model R^2 as a function of the *cis* R^2.

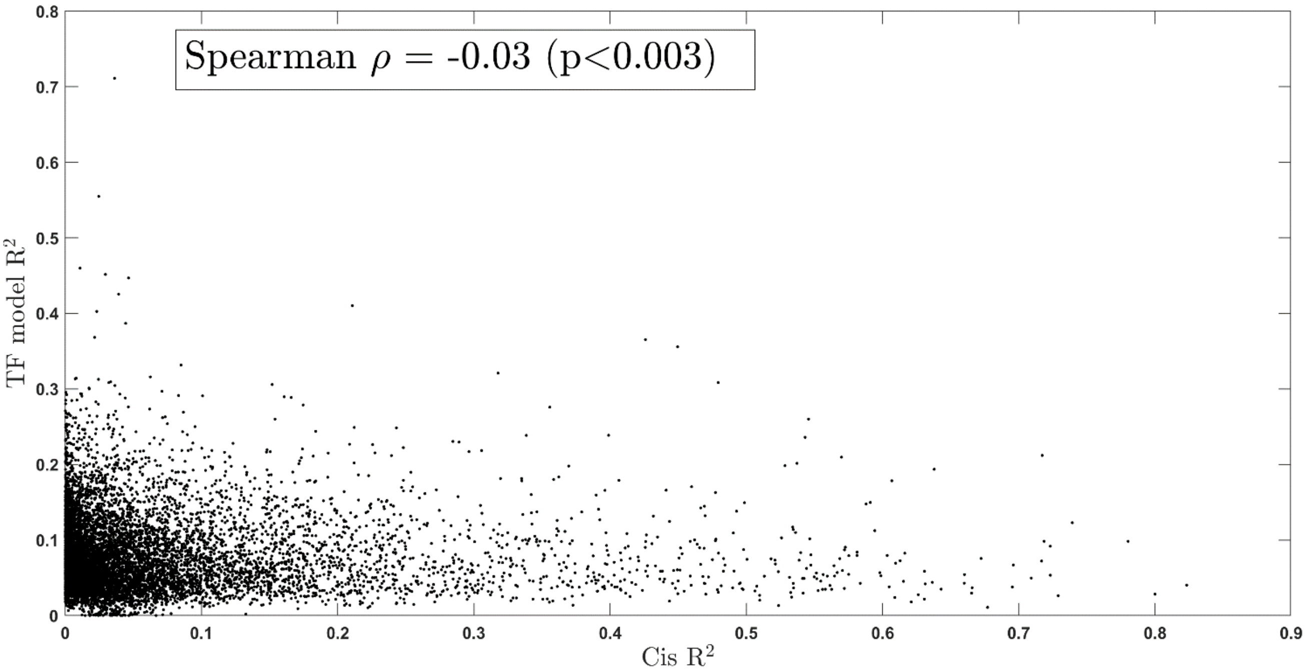

Figure S4. Scatter plot of the TF-Binding model R^2 as a function of the *cis* R^2.

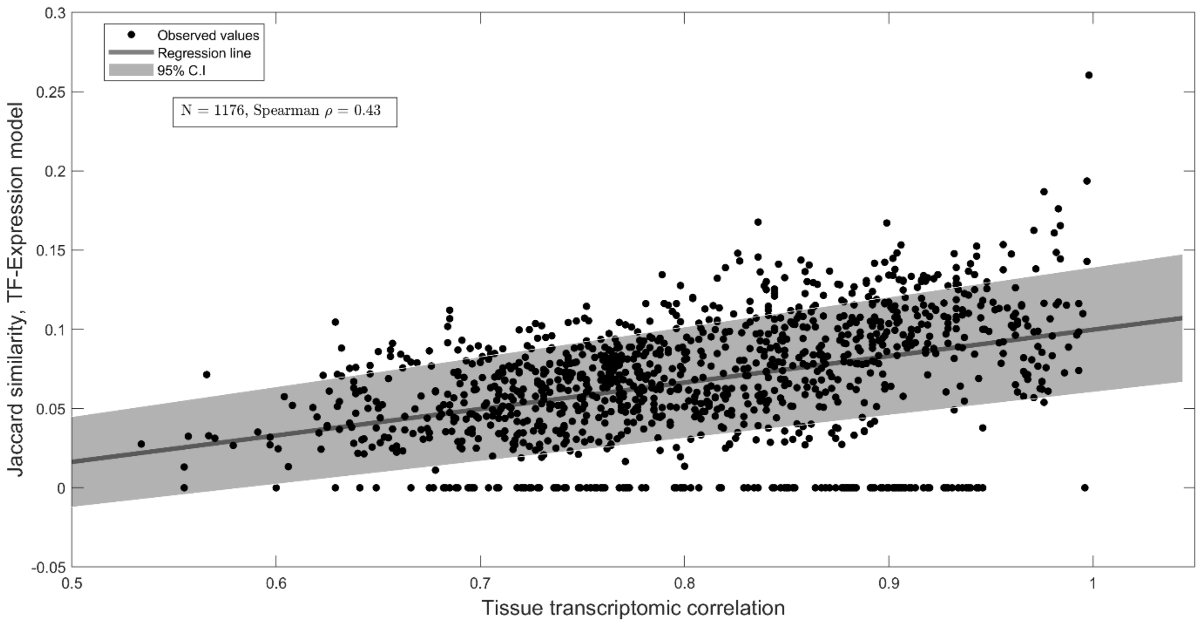

Figure S5. Scatter plot of the Jaccard-based tissue similarity (Y-axis) vs. the transcriptomic correlation similarity from Zhou et al. {Zhou, 2020 #623} for the TF-Expression model.

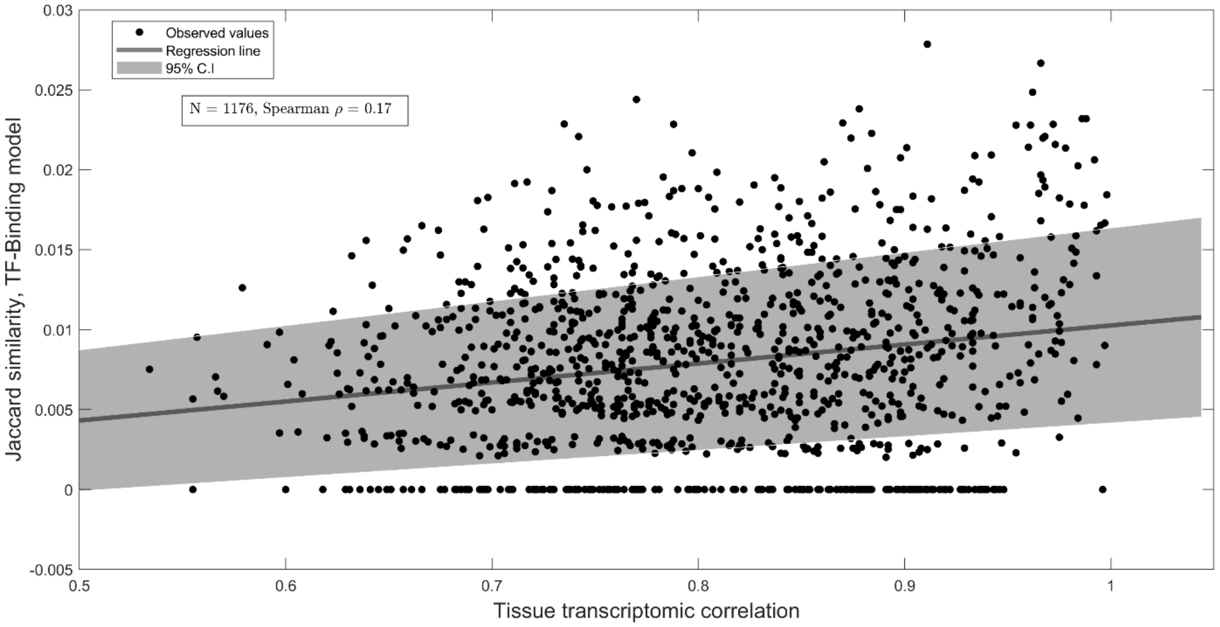

Figure S6. Scatter plot of the Jaccard-based tissue similarity (Y-axis) vs. the transcriptomic correlation similarity from Zhou et al. {Zhou, 2020 #623} for the TF-Binding model.

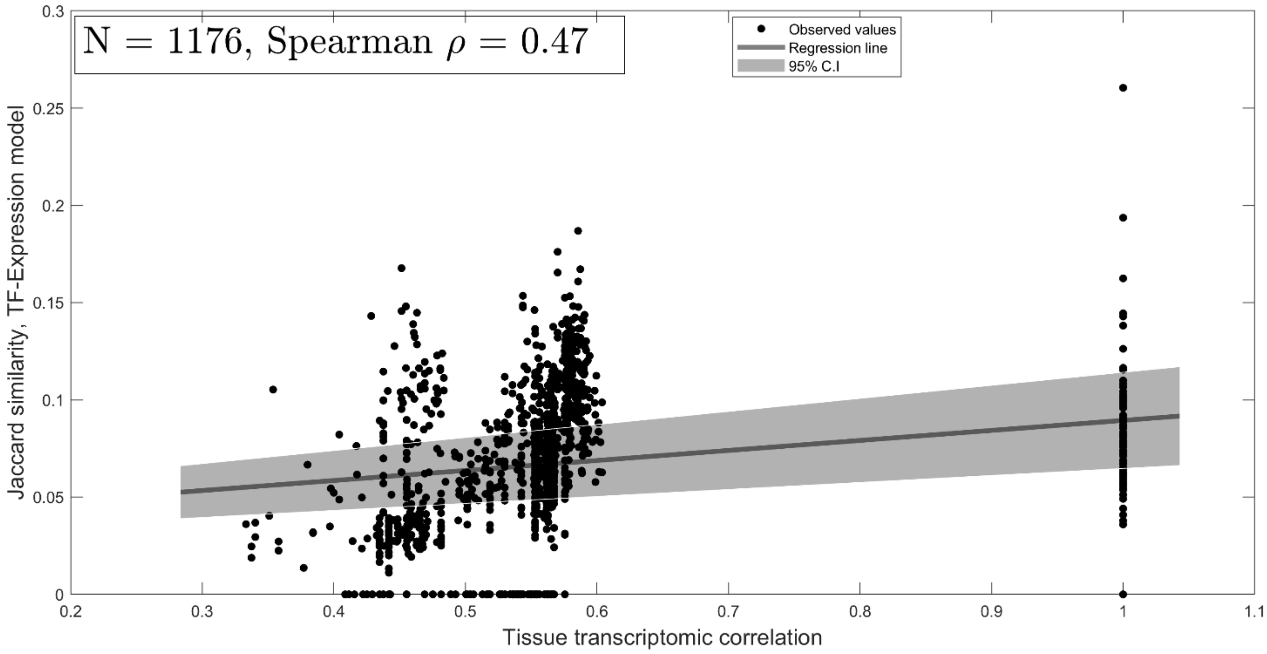

Figure S7. Scatter plot of the Jaccard-based tissue similarity (Y-axis) vs. the DNase I hypersensitive sites (DHS) similarity from Zhou et al. {Zhou, 2020 #623} for the TF-Expression model. The line depicts linear regression line with 95% confidence intervals in gray.

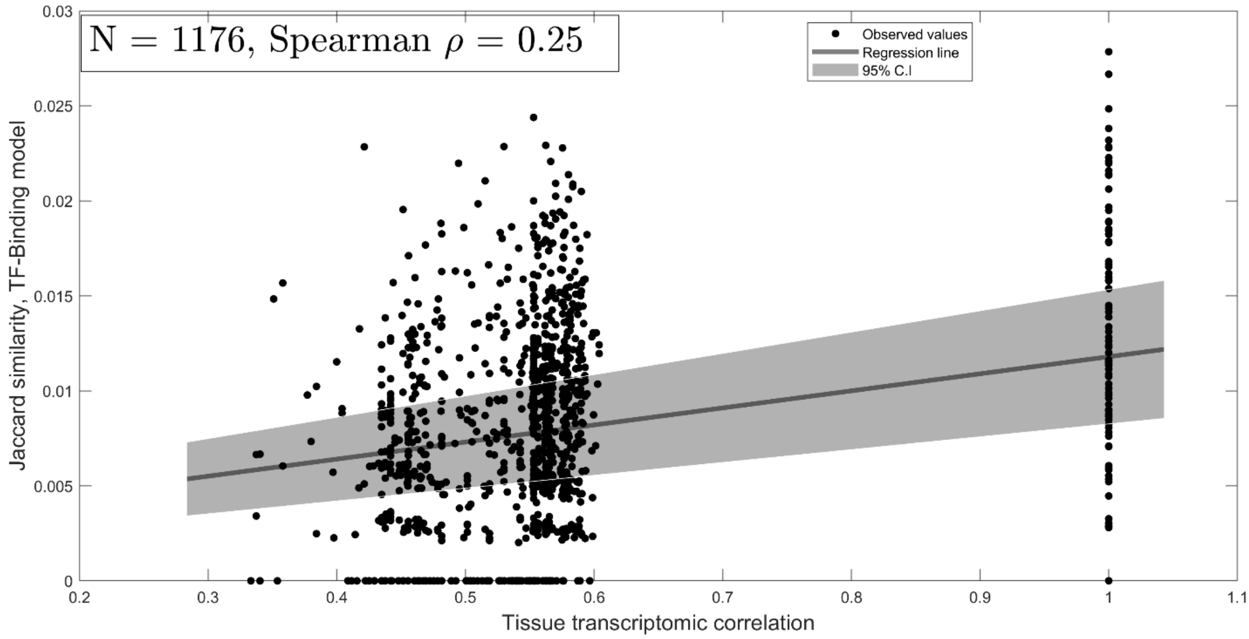

Figure S8. Scatter plot of the Jaccard-based tissue similarity (Y-axis) vs. the DNase I hypersensitive sites (DHS) similarity from Zhou et al. {Zhou, 2020 #623} for the TF-Binding model. The line depicts linear regression line with 95% confidence intervals in gray.

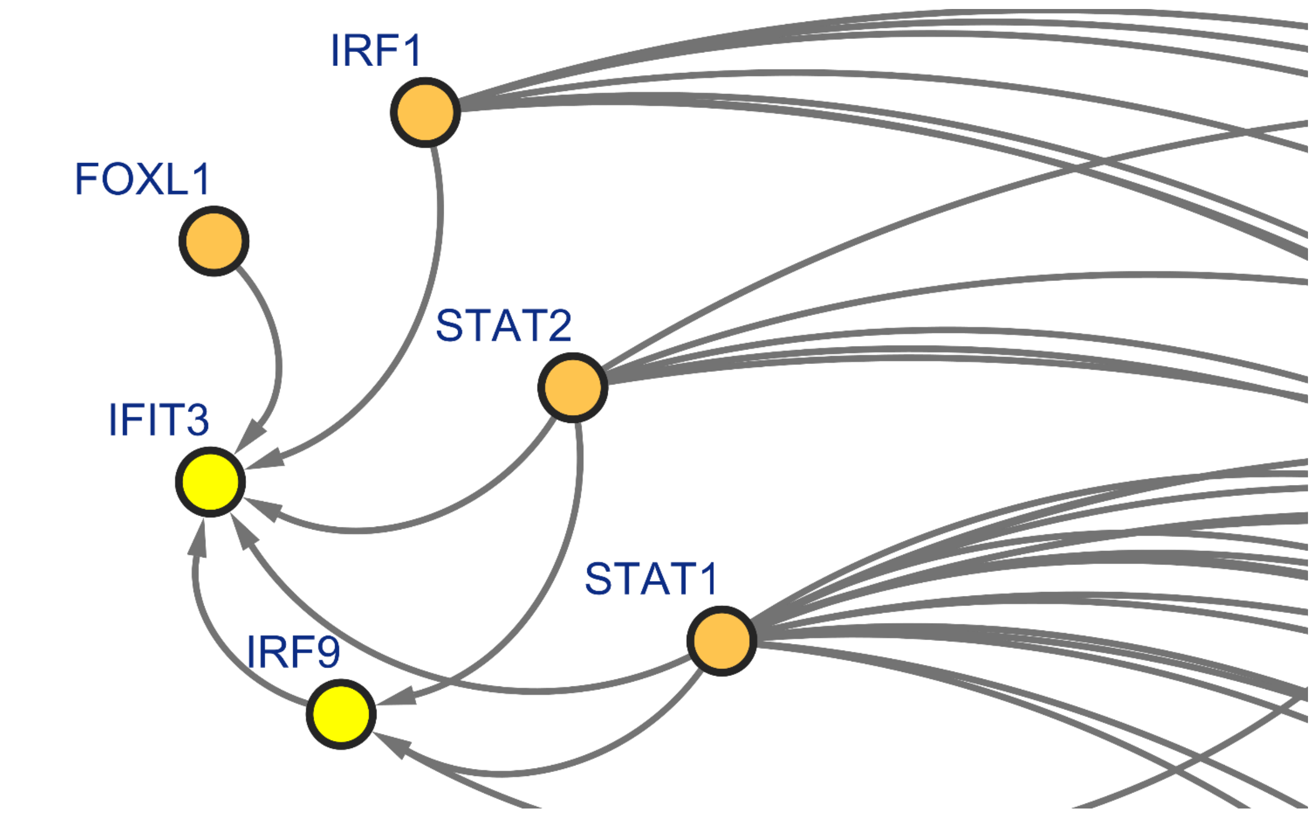

Figure S9. Example of regulation cascade discovered in the TF-Expression model in Amygdala tissue between IFIT3 (hit gene, in yellow), IRF9 (both TF and hit gene, yellow). Other TFs, such as STAT1 and STAT2, are included in the models for IFIT3, IRF9 or both (orange).

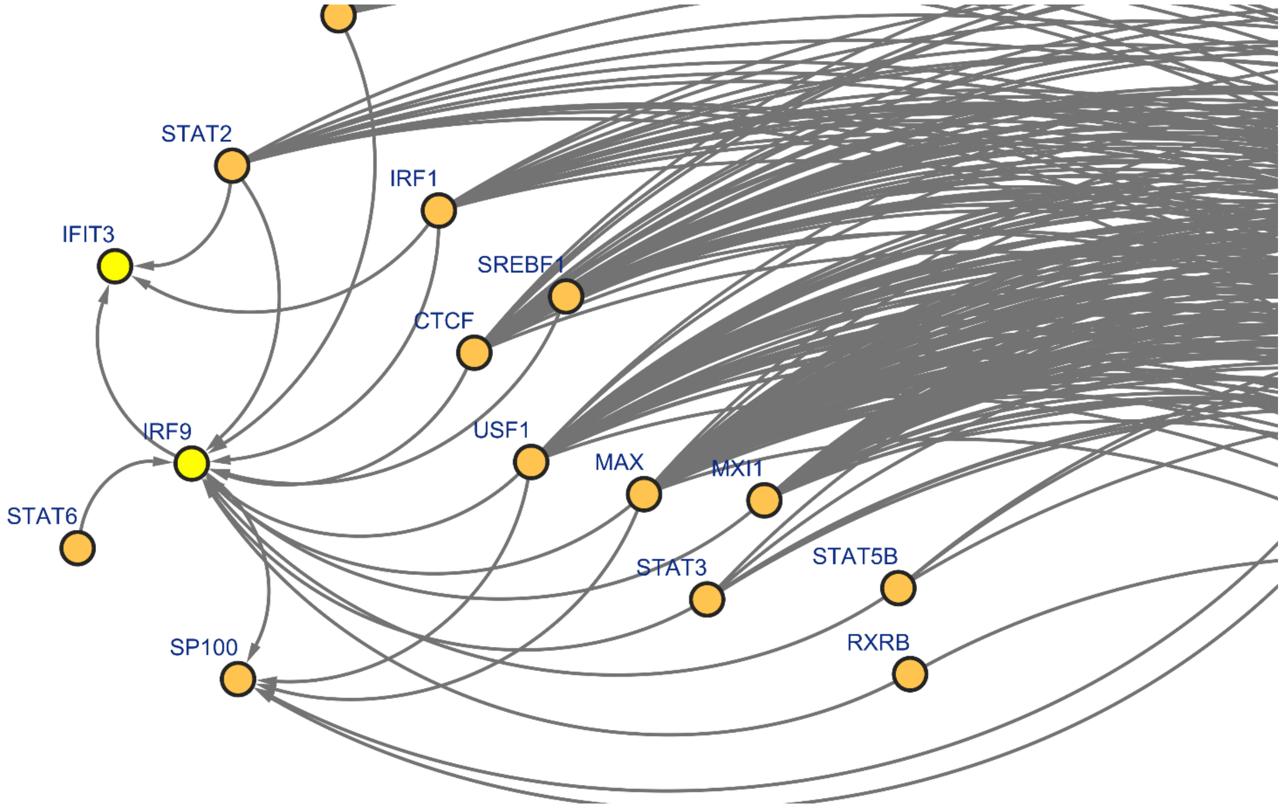

Figure S10. Example of regulation cascade discovered in the TF-Expression model in Esophagus Mucosa tissue between IFIT3 (hit gene, in yellow), IRF9 (both TF and hit gene, yellow). Other TFs, such as STAT2, are included in the models for IFIT3, IRF9 or both (orange).

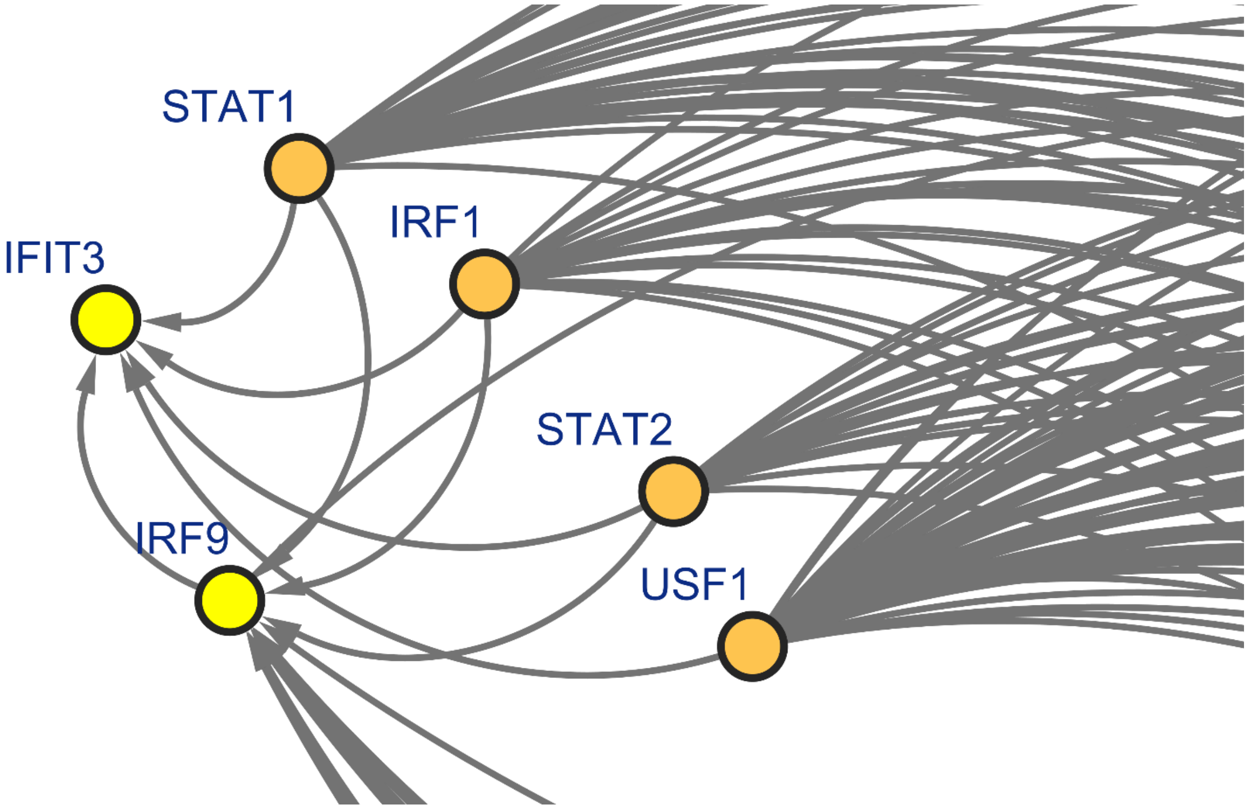

Figure S11. Example of regulation cascade discovered in the TF-Expression model in Skin, sun exposed tissue between IFIT3 (hit gene, in yellow), IRF9 (both TF and hit gene, yellow). Other TFs, such as STAT1, are included in the models for IFIT3, IRF9 or both (orange).

Tables

Table S1. Matrices of Hit Gene x Tissue for the TF-Expression and TF-Binding models. Available as a separate Excel file.

Table S2. Enriched PID and Reactome pathways with TFs participating in the models. Q-values are B&H FDR adjusted p-values.

**TF-Expression**	**q-value**
PID_ALK2_PATHWAY	0.0056
PID_BARD1_PATHWAY	0.0019
PID_GMCSF_PATHWAY	0.0286
PID_HIF1A_PATHWAY	0.0027
PID_IL12_2PATHWAY	0.0071
PID_IL2_1PATHWAY	0.0183
PID_IL27_PATHWAY	0.0170
PID_IL4_2PATHWAY	0.0010
PID_NFKAPPAB_CANONICAL_PATHWAY	0.0084
PID_P38_MK2_PATHWAY	0.0219
PID_RANBP2_PATHWAY	0.0341
PID_TCPTP_PATHWAY	0.0237
PID_TCR_CALCIUM_PATHWAY	0.0015
PID_TGFBR_PATHWAY	0.0056
REACTOME_ABERRANT_REGULATION_OF_MITOTIC_G1_S_TRANSITION_IN_CANCER_DUE_TO_RB1_DEFECTS	0.0077
REACTOME_ACTIVATION_OF_ATR_IN_RESPONSE_TO_REPLICATION_STRESS	0.0337
REACTOME_ACTIVATION_OF_BH3_ONLY_PROTEINS	0.0341
REACTOME_ACTIVATION_OF_THE_PRE_REPLICATIVE_COMPLEX	0.0179
REACTOME_CDC6_ASSOCIATION_WITH_THE_ORC_ORIGIN_COMPLEX	0.0056
REACTOME_CYCLIN_D_ASSOCIATED_EVENTS_IN_G1	0.0056
REACTOME_DDX58_IFIH1_MEDIATED_INDUCTION_OF_INTERFERON_ALPHA_BETA	0.0170
REACTOME_DEFECTIVE_LFNG_CAUSES_SCDO3	0.0197
REACTOME_DEX_H_BOX_HELICASES_ACTIVATE_TYPE_I_IFN_AND_INFLAMMATORY_CYTOKINES_PRODUCTION_	0.0062
REACTOME_DISEASES_OF_SIGNAL_TRANSDUCTION_BY_GROWTH_FACTOR_RECEPTORS_AND_SECOND_MESSENGERS	0.0037
REACTOME_DNA_STRAND_ELONGATION	0.0152
REACTOME_DSCAM_INTERACTIONS	0.0341
REACTOME_E2F_MEDIATED_REGULATION_OF_DNA_REPLICATION	0.0273
REACTOME_FGFR1_MUTANT_RECEPTOR_ACTIVATION	0.0127

REACTOME_FORMATION_OF_INCISION_COMPLEX_IN_GG_NER	0.0273
REACTOME_FORMATION_OF_THE_BETA_CATENIN_TCF_TRANSACTIVATING_COMPLEX	0.0284
REACTOME_GENOME_REPLICATION_AND_TRANSCRIPTION	0.0339
REACTOME_INTERFERON_GAMMA_SIGNALING	0.0147
REACTOME_INTERLEUKIN_4_AND_INTERLEUKIN_13_SIGNALING	0.0013
REACTOME_INTERLEUKIN_9_SIGNALING	0.0172
REACTOME_MITOTIC_G1_PHASE_AND_G1_S_TRANSITION	0.0023
REACTOME_NF_KB_IS_ACTIVATED_AND_SIGNALS_SURVIVAL	0.0122
REACTOME_NR1H2_AND_NR1H3_MEDIATED_SIGNALING	0.0172
REACTOME_NR1H3_NR1H2_REGULATE_GENE_EXPRESSION_LINKED_TO_CHOLESTEROL_TRANSPORT_AND_EFFLUX	0.0337
REACTOME_NUCLEOTIDE_BINDING_DOMAIN_LEUCINE_RICH_REPEAT_CONTAINING_RECEPTOR_NLR_SIGNALING_PATHWAYS	0.0459
REACTOME_OXIDATIVE_STRESS_INDUCED_SENESCENCE	0.0259
REACTOME_P75NTR_SIGNALS_VIA_NF_KB	0.0284
REACTOME_PRE_NOTCH_PROCESSING_IN_THE_ENDOPLASMIC_RETICULUM	0.0339
REACTOME_PTK6_EXPRESSION	0.0197
REACTOME_RESOLUTION_OF_D_LOOP_STRUCTURES	0.0209
REACTOME_SIGNALING_BY_BMP	0.0066
REACTOME_SIGNALING_BY_FGFR1_IN_DISEASE	0.0133
REACTOME_SIGNALING_BY_NOTCH	0.0003
REACTOME_STING_MEDIATED_INDUCTION_OF_HOST_IMMUNE_RESPONSES	0.0056
REACTOME_SUMOYLATION_OF_CHROMATIN_ORGANIZATION_PROTEINS	0.0432
REACTOME_TAK1_ACTIVATES_NFKB_BY_PHOSPHORYLATION_AND_ACTIVATION_OF_IKKS_COMPLEX	0.0474
REACTOME_TFAP2_AP_2_FAMILY_REGULATES_TRANSCRIPTION_OF_GROWTH_FACTORS_AND_THEIR_RECEPTORS	0.0218
REACTOME_TFAP2A_ACTS_AS_A_TRANSCRIPTIONAL_REPRESSOR_DURING_RETINOIC_ACID_INDUCED_CELL_DIFFERENTIATION	0.0197
REACTOME_TOLL_LIKE_RECEPTOR_TLR1_TLR2_CASCADE	0.0207
REACTOME_TP53_REGULATES_TRANSCRIPTION_OF_CASPASE_ACTIVATORS_AND_CASPASES	0.0468
REACTOME_TP53_REGULATES_TRANSCRIPTION_OF_GENES_INVOLVED_IN_CYTOCHROME_C_RELEASE	0.0178
REACTOME_TRAF3_DEPENDENT_IRF_ACTIVATION_PATHWAY	0.0169
REACTOME_UNFOLDED_PROTEIN_RESPONSE_UPR_	0.0134
**TF-Binding**	**q-value**
PID_GMCSF_PATHWAY	0.0447

PID_NFKAPPAB_CANONICAL_PATHWAY	0.0042
PID_IL12_2PATHWAY	0.0460
PID_ATM_PATHWAY	0.0340
PID_IL2_PI3K_PATHWAY	0.0340
PID_HIF1A_PATHWAY	0.0348
PID_IL2_STAT5_PATHWAY	0.0182
PID_TCR_CALCIUM_PATHWAY	0.0454
PID_BARD1_PATHWAY	0.0039
PID_TGFBR_PATHWAY	0.0469
REACTOME_SIGNALING_BY_NOTCH	0.0018
REACTOME_DDX58_IFIH1_MEDIATED_INDUCTION_OF_INTERFERON_ALPHA_BETA	0.0143
REACTOME_STING_MEDIATED_INDUCTION_OF_HOST_IMMUNE_RESPONSES	0.0031
REACTOME_PRE_NOTCH_PROCESSING_IN_THE_ENDOPLASMIC_RETICULUM	0.0254
REACTOME_NF_KB_IS_ACTIVATED_AND_SIGNALS_SURVIVAL	0.0406
REACTOME_ENDOGENOUS_STEROLS	0.0388
REACTOME_DEX_H_BOX_HELICASES_ACTIVATE_TYPE_I_IFN_AND_INFLAMMATORY_CYTOKINES_PRODUCTION_	0.0041
REACTOME_IRF3_MEDIATED_INDUCTION_OF_TYPE_I_IFN	0.0406
REACTOME_TAK1_ACTIVATES_NFKB_BY_PHOSPHORYLATION_AND_ACTIVATION_OF_IKKS_COMPLEX	0.0254
REACTOME_MITOTIC_G1_PHASE_AND_G1_S_TRANSITION	0.0179
REACTOME_DEFECTIVE_LFNG_CAUSES_SCDO3	0.0148
REACTOME_B_WICH_COMPLEX_POSITIVELY_REGULATES_RRNA_EXPRESSION	0.0012
REACTOME_DISEASES_OF_IMMUNE_SYSTEM	0.0235
REACTOME_GLI_PROTEINS_BIND_PROMOTERS_OF_HH_RESPONSIVE_GENES_TO_PROMOTE_TRANSCRIPTION	0.0386
REACTOME_DISEASES_OF_SIGNAL_TRANSDUCTION_BY_GROWTH_FACTOR_RECEPTORS_AND_SECOND_MESSENGERS	0.0055
REACTOME_DNA_DOUBLE_STRAND_BREAK_REPAIR	0.0485
REACTOME_DNA_DOUBLE_STRAND_BREAK_RESPONSE	0.0128
REACTOME_INTERLEUKIN_4_AND_INTERLEUKIN_13_SIGNALING	0.0208
REACTOME_TP53_REGULATES_TRANSCRIPTION_OF_GENES_INVOLVED_IN_CYTOCHROME_C_RELEASE	0.0422
REACTOME_TP53_REGULATES_TRANSCRIPTION_OF_CASPASE_ACTIVATORS_AND_CASPASES	0.0313
REACTOME_CDC6_ASSOCIATION_WITH_THE_ORC_ORIGIN_COMPLEX	0.0235
REACTOME_ACTIVATION_OF_THE_PRE_REPLICATIVE_COMPLEX	0.0296

REACTOME_DNA_STRAND_ELONGATION	0.0254
REACTOME_PTK6_EXPRESSION	0.0148
REACTOME_TFAP2A_ACTS_AS_A_TRANSCRIPTIONAL_REPRESSOR_DURING_RETINOIC_ACID_INDUCED_CELL_DIFFERENTIATION	0.0148
REACTOME_ARYL_HYDROCARBON_RECEPTOR_SIGNALLING	0.0386
REACTOME_RUNX1_REGULATES_TRANSCRIPTION_OF_GENES_INVOLVED_IN_DIFFERENTIATION_OF_MYELOID_CELLS	0.0386
REACTOME_INTERLEUKIN_15_SIGNALING	0.0100
REACTOME_NR1H2_AND_NR1H3_MEDIATED_SIGNALING	0.0021
REACTOME_NR1H3_NR1H2_REGULATE_GENE_EXPRESSION_LINKED_TO_CHOLESTEROL_TRANSPORT_AND_EFFLUX	0.0046
REACTOME_TRAF3_DEPENDENT_IRF_ACTIVATION_PATHWAY	0.0100

Table S3. The list of tissues and cell used in the study.

Adipose Subcutaneous	Esophagus Mucosa
Adipose Visceral Omentum	Esophagus Muscularis
Adrenal Gland	Heart Atrial Appendage
Artery Aorta	Heart Left Ventricle
Artery Coronary	Kidney Cortex
Artery Tibial	Liver
Brain Amygdala	Lung
Brain Anterior cingulate cortex BA24	Minor Salivary Gland
Brain Caudate basal ganglia	Nerve Tibial
Brain Cerebellar Hemisphere	Ovary
Brain Cerebellum	Pancreas
Brain Cortex	Pituitary
Brain Frontal Cortex BA9	Prostate
Brain Hippocampus	Skin Not Sun Exposed Suprapubic
Brain Hypothalamus	Skin Sun Exposed Lower leg
Brain Nucleus accumbens basal ganglia	Small Intestine Terminal Ileum
Brain Putamen basal ganglia	Spleen
Brain Spinal cord cervical c-1	Stomach
Brain Substantia nigra	Testis
Breast Mammary Tissue	Thyroid
Cells Cultured fibroblasts	Uterus
Cells EBV-transformed lymphocytes	Vagina
Colon Sigmoid	Muscle Skeletal
Colon Transverse	Whole Blood
Esophagus Gastroesophageal Junction	
